# Prediction of amyloid fibril-forming segments based on a support vector machine

**DOI:** 10.1186/1471-2105-10-S1-S45

**Published:** 2009-01-30

**Authors:** Jian Tian, Ningfeng Wu, Jun Guo, Yunliu Fan

**Affiliations:** 1 Biotechnology Research Institute, Chinese Academy of Agricultural Sciences, Beijing 100081, PR China

## Abstract

**Background:**

Amyloid fibrillar aggregates of proteins or polypeptides are known to be associated with many human diseases. Recent studies suggest that short protein regions trigger this aggregation. Thus, identifying these short peptides is critical for understanding diseases and finding potential therapeutic targets.

**Results:**

We propose a method, named Pafig (Prediction of amyloid fibril-forming segments) based on support vector machines, to identify the hexpeptides associated with amyloid fibrillar aggregates. The features of Pafig were obtained by a two-round selection from AAindex. Using a 10-fold cross validation test on Hexpepset dataset, Pafig performed well with regards to overall accuracy of 81% and Matthews correlation coefficient of 0.63. Pafig was used to predict the potential fibril-forming hexpeptides in all of the 64,000,000 hexpeptides. As a result, approximately 5.08% of hexpeptides showed a high aggregation propensity. In the predicted fibril-forming hexpeptides, the amino acids – alanine, phenylalanine, isoleucine, leucine and valine occurred at the higher frequencies and the amino acids – aspartic acid, glutamic acid, histidine, lysine, arginine and praline, appeared with lower frequencies.

**Conclusion:**

The performance of Pafig indicates that it is a powerful tool for identifying the hexpeptides associated with fibrillar aggregates and will be useful for large-scale analysis of proteomic data.

## Background

Understanding protein aggregation has become increasingly important with the discovery of a correlation between amyloid-like fibrils resulting from protein aggregation and diseases such as Alzheimer's disease, Parkinson's disease, transmissible spongiform encephalopathies, and type II diabetes [1-4].

Not only the intrinsically disordered proteins, but also natively folded proteins can aggregate into amyloid-like fibrils, as has been found in β2-microglobulin, lysozyme, transthyretin, and the prion protein [5-7]. It is believed that specific continuous regions within the amyloid fibril-forming proteins can act as facilitators or inhibitors of amyloid fibril formation and determine the proteins' aggregation tendency [6,8-12]. Therefore, recognizing the specific regions that determine the aggregation propensity of a protein is of fundamental interest, since this will help in understanding the mechanism of amyloid formation and lead to effective treatments for amyloid illnesses [13].

As reviewed recently [13], there are two types of computational approaches used to investigate the aggregation propensity of peptides or proteins and to identify the segments most prone to form fibrils (hot spots). The first approach uses phenomenological models based on the physicochemical properties of the amino acids (e.g. β-propensity, hydrophobicity, aromatic content, and charge) to predict changes in aggregation rates with mutation as well as absolute aggregation rates and hot spots [5,6,14-19]. The second approach combines atomistic simulations of a protein segment with the microcrystal structure of short fibril-forming peptides to gain insight into aggregation propensity [1,20-22]. This approach may help to elucidate the structural details of ordered aggregates. In addition to the approaches described above, a sequence pattern obtained by saturation mutagenesis analysis [11] has been proposed to identify amyloidogenic stretches in proteins.

In this study, we proposed the Pafig (Prediction of amyloid fibril-forming segments) to identify fibril-forming segments in proteins based on a support vector machine (SVM) [23,24]. The predictive model of Pafig was also a phenomenological model, which was based on 41 physicochemical properties selected by a two-round selection from 531 physicochemical properties in the Amino acid index database (AAindex) [25,26]. Because short regions of a protein were responsible for its amyloidogenic behavior [1,5,6,11,18], Pafig was trained by hexpeptides, which were decomposed by scanning for segments that could form fibrils with a six-residue sliding window. Pafig is simple, fast, and suitable for large-scale calculations such as proteomics analyses.

## Methods

### Datasets and physicochemical properties

In this study, we constructed a dataset, the Hexpepset dataset, to train the model and test the robustness of Pafig. The six-residue peptides that could form fibrils were defined as positive samples and those that could not form fibrils as negative samples. The Hexpepset dataset consisted of 2452 hexpeptides (1226 positive samples and 1226 negative samples). The positive samples in the Hexpepset dataset were collected by scanning known fibril-forming fragments [1,5,6,8,14,18,20,27] (Additional file 1) with a six-residue window. Because a large difference between the positive and negative samples can hamper training of the SVM [28,29], the negative set also contained 1226 hexpeptides selected from two different sources. The first negative sample part contained 876 samples, which was selected by scanning the fragments that had been proved not to form fibrils by experiments[18,20]. The second negative sample part consisted of 350 samples. These samples were randomly selected from the hexpeptides obtained by scanning the fragments except the experimentally determined amyloidogenic regions of five proteins (Transthyretin, Genbank Accession No. AAB35640.1; Major prion protein precursor, Genbank Accession No. P04156; Apo-AI, Genbank Accession No. P02647; Alpha-synuclein, Genbank Accession No. P37840.1; Beta-2-Microglobulin, Genbank Accession No. 1LDS) [1,6,27]. The Hexpepset dataset could be downloaded from website of Pafig [30].

There are totally 544 physicochemical properties in the amino acid index database version 9.0 (AAindex) [25,26], which is a collection of published amino acid indices representing different physicochemical and biological properties of amino acids. Each physicochemical property consists of a set of 20 numerical values for amino acids. The property having the value 'NA' in a value set of amino acid index was discarded. Finally, 531 properties were used for the following mining method.

### Physicochemical properties selection and Encoding Schemes of Pafig

**Figure 1:**
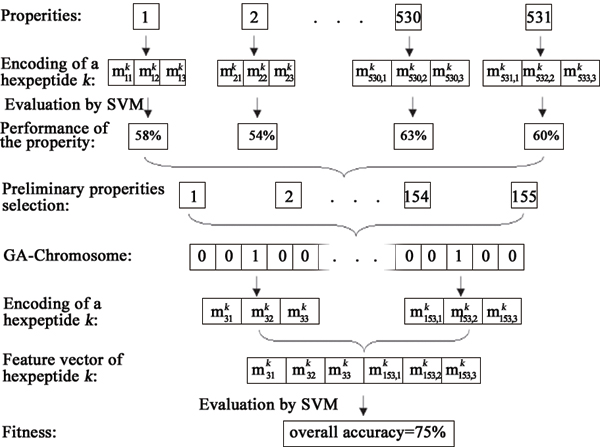
Physicochemical property selection and encoding schemes of Pafig.

The input feature of Pafig consisted of 123 elements based on 41 physicochemical properties. The properties selection and encoding schemes of Pafig was illustrated in Figure 1 and calculated as follows:

The 531 physicochemical properties were downloaded from Aaindex [25]. The values of each property were scaled to zero mean and a standard deviation of 1.

According to a physicochemical property *p_i_*, a hexpeptide *h^k^* could be mapped to feature vector *F_i_* with three elements. $F_{i}^{k}=[m_{i1}^{k},m_{i2}^{k},m_{i3}^{k}]$, $m_{i1}^{k}=\sum_{n=1}^{n=6}{\left[x_{i}^{k}(n)\right]}^{2}$, $m_{i2}^{k}=\sum_{n=1}^{n=6}{\left[x_{i}^{k}(n)\right]}^{2}\times \delta (n)$. If the value of $x_{i}^{k}(n)$ was greater than zero, the value of δ(n) is 1; otherwise it was 0. $m_{i3}^{k}=\sum_{n=1}^{n=6}{\left[x_{i}^{k}(n)\right]}^{2}\times \delta (n)$. If the value of $x_{i}^{k}(n)$ was smaller than zero, the value of δ(n) was 1; otherwise it was 0. Here $x_{i}^{k}(n)$ was one of physicochemical property corresponding to the n^th^ amino acid.

The Support vector machine was used to evaluate the classification ability of each property. The property, which obtained the overall accuracy greater or equal than 60%, was selected as the candidate of Pafig.

A standard genetic algorithm [31] was used to select the final physicochemical properties of Pafig with population size of 10, crossover probability of 0.8, mutation probability of 0.01 and predetermined number of 200 generations. The overall accuracy (Q2) was adopted as the fitness.

### Support vector machine

Support vector machine (SVM) was used to identify an optimal hyperplane in order to separate two classes of samples [23,24]. SVM uses kernel functions to map the original data to a feature space of higher dimensions and also locates an optimal separating hyperplane. For SVM implementation, we used LIBSVM [32] with a Radial Basis Function (RBF kernel) and a Polynomial kernel. The parameters were selected with the LIBSVM parameter selection tool (easy.py).

### Prediction system assessment

True positives (TP) and true negatives (TN) were identified as the positive and negative samples, respectively. False positives (FP) were negative samples identified as positive. False negatives (FN) were positive samples identified as negative. The prediction performance was tested with sensitivity (TP/(TP+FN)), specificity (TN/(TN+FP)), overall accuracy (Q2), and the Matthews correlation coefficient (MCC). The Q2 and MCC were calculated as follows:

$$\begin{array}{l} MCC=\frac{TP\times TN-FP\times FN}{\sqrt{\left(TN+FN)\times (TN+FP)\times (TP+FN)\times (TP+FP\right)}}\\ Q2=\frac{TP+TN}{TP+TN+FP+FN} \end{array}$$

## Results

### Properties selection

**Figure 2:**
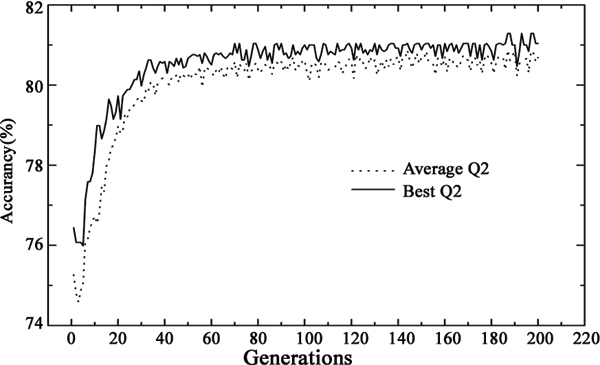
Average overall accuracy among the individuals and the best overall accuracy of different generations. The results were obtained by LIBSVM with 10-cross validation on Hexpepset dataset. Q2: overall accuracy.

The properties of Pafig were selected by a two-round selection on Hexpepset dataset from the AAindex [25,26]. Firstly, a preliminary properties selection on all of the physicochemical properties (531 properties) was performed using the following steps. (1) Every hexpeptide in the Hexpepset dataset was encoded by each physicochemical property. (2) The overall prediction accuracy (Q2) corresponding to each property was calculated by LIBSVM with a 10-Cross validation on Hexpepset dataset. (3) All performance of each physicochemical property was ranked according to their overall prediction accuracy (Q2). (4) The overall prediction accuracy (Q2 = 60%) was used as the cut off to select properties. As a result, 155 physicochemical properties were selected from 531 properties in AAindex and used as the candidates for further selection. Secondly, an efficient bi-objective genetic algorithm with population size of 10, crossover probability of 0.8, mutation probability of 0.01 and predetermined number of 200 generations was utilized to mine informative physicochemical properties and combine the selected properties to build a prediction model. The overall prediction accuracy (Q2) was adopted as the fitness. All results of genetic algorithm were obtained by LIBSVM with a 10-Cross validation on Hexpepset dataset and were shown in Figure 2.

As shown in Figure 2, the SVM classifier obtained the best overall accuracy Q2 = 81% and MCC = 0.63 in an individual of the 186^th^ generation, where the number of selected physicochemical properties was 41. The standard deviations of Q2 and MCC among the 10-fold cross-validation of the best individual were 1% and 0.01, respectively. Table 1 shows the selected physicochemical properties by Genetic Algorithm and the overall accuracy obtained by the corresponding property. The best property of all the selected properties was VINM940104, which obtained the best overall accuracy Q2 = 66% and corresponds to the "Normalized flexibility parameters (B-values) for each residue surrounded by two rigid neighbours" [33].

**Table 1:** The AAindex identities and the overall accuracy (Q2) of the 41 properties selected by Genetic Algorithm. The overall prediction accuracy (Q2) was calculated using the corresponding property by LIBSVM with RBF kernel of 10-Cross validation on Hexpepset dataset.

ID of AAindex	Q2	ID of AAindex	Q2
VINM940104	66	CHOC760104	62
JACR890101	64	CORJ870103	62
NADH010103	63	CHOP780211	62
MONM990201	63	EISD860101	62
DESM900101	63	CIDH920104	62
JANJ780103	63	NAKH920105	62
ROSM880102	63	WOLR810101	62
PONP800102	63	ZIMJ680103	61
NADH010104	63	PARS000101	61
MEIH800102	62	KIMC930101	61
VINM940103	62	NAKH900108	61
RACS770102	62	CORJ870106	61
BEGF750102	62	RICJ880113	60
CHOP780210	62	KOEP990102	60
MONM990101	62	COWR900101	60
BAEK050101	62	GUYH850102	60
OLSK800101	62	NADH010105	60
PONP930101	62	WERD780101	60
PALJ810104	62	CHAM830101	60
FASG890101	62	RACS770101	60
MIYS990105	62		

### Prediction performance of Pafig

We investigated the performances using 41, 155, or 531 physicochemical properties. The 10-fold cross validation tests on the Hexpepset dataset were carried out by LIBSVM with RBF kernel. As shown in Table 2, the classifier employing the 41 properties obtained the best performance, with the overall prediction accuracy (Q2) of 81% and the Matthews correlation coefficient (MCC) of 0.63, which was better than using 155 and 531 physicochemical properties. This improvement is mainly due to the reduction in noise and outliers present in the 378 and 531 physicochemical properties, which influence the performance of the Support Vector Machine [34]. In addition, we also evaluated the effect of different SVM kernel and found that the performance with the Radial Basis Function (RBF) kernel was better than the Polynomial kernel, which only obtained an overall accuracy (Q2) of 77% and a Matthews correlation coefficient (MCC) of 0.54 using the selected 41 physicochemical properties with best parameter of d = 3.

**Table 2:** Prediction performance on the Hexpepset dataset with different properties. All of the results were obtained by LIBSVM with RBF kernel.

Number of properties	Specificity(%)	Sensitivity(%)	Q2^a ^(%)	MCC^b^
1^c^	61	70	66	0.31
41^d^	80	82	81	0.63
155^e^	71	73	72	0.45
531^f^	98	15	57	0.24

^a^overall prediction accuracy; ^b^MCC, Matthews correlation coefficient; ^c^the property of VINM940104; ^d^properties selected by Genetic Algorithm; ^e^properties that obtained the Q2 higher or equal than 60%; ^f^all physicochemical properties in the AAindex.

**Figure 3:**
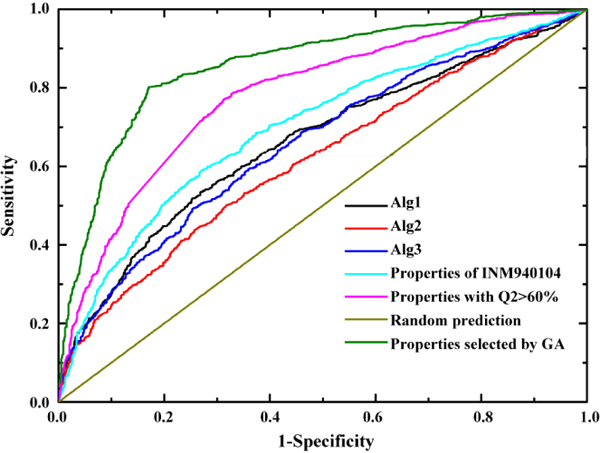
ROC curves of random classifier, classifier with property of VINM940104, properties with overall prediction accuracy (Q2) ≥ 60% and properties selected by Genetic Algorithm and some published classifiers (Alg1 [27] Alg2 [5] and Alg3 [6]).

The receiver operating characteristics (ROC) score was usually used as the primary measure of the machine learning method performance and provided an overview of the possible cut-off levels in the test performance [35]. The ROC curves for the random classifier and classifiers with property of VINM940104, properties with overall prediction accuracy (Q2) ≥ 60% and properties selected by Genetic Algorithm were shown in Figure 3. The result revealed that the classifier with the 41 predictive properties selected by genetic algorithm was better than the property of VINM940104 and properties with overall prediction accuracy (Q2) ≥ 60%.

In addition, we also compared the performance of Pafig with other methods [5,6,27] using the same dataset. The Alg1 [27] is based on the intrinsic aggregation propensities to identify the regions of the protein sequence that are most important for promoting amyloid formation. The Alg2 [5] and Alg3 [6] were used the observed packing density and the relative experimental aggregation propensities of the 20 natural amino acids to detect the amyloid fibril-forming segments. As shown in the Figure 3, we could clearly found that performance of Pafig was better than these popular methods. Moreover, these results also indicate that Pafig is a powerful tool for predicting the amyloid fibril-forming segments in the protein sequence.

### Reliability index for Pafig predictions

**Figure 4:**
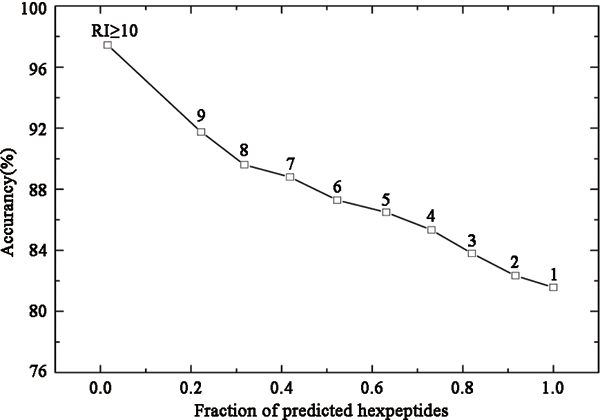
**Average prediction accuracy calculated cumulatively with RI above a given value**. For example, 42% of all hexpeptides had an RI ≥ 7, and of these hexpeptides, 89% were correctly predicted. The results were based on the Hexpepset dataset with 10-fold cross validations.

When machine learning approaches are selected to classify the samples, it is important to know the reliability of the prediction result [36-39]. In this study, a reliability index (RI) was assigned to a predicted hexpeptide based on the output of LIBSVM. Provided that an output of LIBSVM for a hexpeptide is O, the RI value is thus computed as RI = INTEGER (20 × abs(O-0.5)). The value of RI could provide information about the certainty of the classification and could be used as an indicator of prediction certainty for a particular hexpeptide. Figure 4 showed the expected prediction accuracies and the fraction of hexpeptides with a given RI value. For example, about 42% of the hexpeptides had an RI ≥ 7, and of these, 89% were correctly predicted. The result was obtained by LIBSVM with 10-fold cross validations on the Hexpepset dataset.

### Identification of fibril forming peptides in all hexpeptides

**Figure 5:**
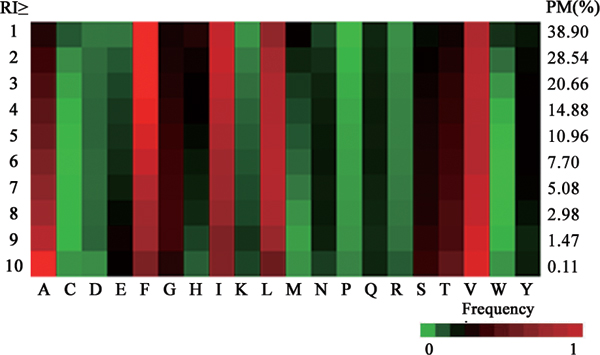
**The frequency of amino acid in the predicted fibril-forming hexpeptides with different RI cutoffs**. The left column is the different RI cutoffs. PM(%) in the right column is the percentage of predicted fibril-forming hexpeptides in all hexpeptides. The other columns represent the frequency of different amino acids in the predicted fibril-forming hexpeptides with different RI cutoffs. Red represents higher frequency, green represents lower frequency and black represents equal levels of frequency.

Pafig was used to predict the potential fibril-forming hexpeptides in all of the 64,000,000 hexpeptides. The fraction of possible fibril-forming hexpeptides with different RI cutoffs was shown in Figure 5. We found that 5.08% of fibril-forming hexpeptides had an RI ≥ 7, which was the minority of all hexpeptides [11]. As shown in Figure 5, the hydrophobic amino acids (alanine, phenylalanine, isoleucine, leucine and valine) occurred at the higher frequencies in the predicted fibril formation hexpeptides. These results matched the hypothesis [6,27,40] that hydrophobic residues usually induced aggregation. However, the amino acids with positive or negative charges, such as aspartic acid, glutamic acid, histidine, lysine and arginine appeared in the predicted fibril formation hexpeptides with lower frequencies. In addition, proline was found with the lowest frequency in the predicted fibril-forming hexpeptides, as expected, as proline was a β-sheet breaker and most fibrils had crossing β-structures [11,41].

### Identification of the frequency of fibril-forming hexpeptides in proteins of the UniProt database

The UniProt Knowledgebase (Release 13.5) analysed here was taken from the European Bioinformatics Institute (EBI), since it was the central access point for extensively curated protein information, including function, classification and cross-references. Pafig was used to predict the possible fibril forming hexpeptides of every protein in the database. As shown in Table 3, archaea, bacteria and plants had a consistently higher frequency of fibril-forming hexpeptides. However, human had a relatively lower aggregation propensity. These results also suggested that evolution was as a factor to oppose protein aggregation by minimizing the amount of strongly aggregating sequence stretches [42]. Moreover, we classified the UniProt Knowledgebase based on the gene ontology [43]. As shown in Table 4, the frequency of fibril forming hexpeptides was very similar among the gene ontology Biological process, Cellular component and Molecular function classes. However, the sub-classes of GO:0009055(electron carrier activity) and GO:0005215(transporter activity) in the molecular function class, which consisted of many membrane proteins, had higher frequency of fibril-forming hexpeptides. This result indicated that the aggregation propensity of membrane proteins was somewhat higher than other proteins.

**Table 3:** Fibril-forming Propensity of proteins in the Uniprot Knowledgebase classed based on taxonomy. The Predicted fibril-forming hexpeptides were identified by obtaining RI ≥ 7.

Taxonomic divisions^a^	Number of Proteins	Number of hexpeptides	Frequency of Predicted fibril-forming hexpeptides
Archaea	14617	4124738	0.12 ± 0.18
Bacteria	222363	68121983	0.12 ± 0.18
Fungi	21711	10261229	0.11 ± 0.16
Human	19804	10726378	0.09 ± 0.14
Invertebrates	16589	6276047	0.10 ± 0.15
Mammals	18319	6547411	0.11 ± 0.16
Plants	25129	8265929	0.12 ± 0.19
Rodents	24350	12441185	0.10 ± 0.14
Vertebrates	13852	4709586	0.10 ± 0.15
Viruses	12283	5518406	0.10 ± 0.15

^a^The division of proteins is same to the Uniprot database. Every protein is present in exactly one taxonomic division. Human contains all human related proteins. Mammals contain all mammalian proteins except those from human and rodents. Vertebrates contain all vertebrate proteins except those from mammals. Invertebrates contain all eukaryotic proteins except those from vertebrates, fungi and plants.

**Table 4:** Fibril-forming Propensity of proteins in the Uniprot Knowledgebase classed based on Gene Ontology. The Predicted fibril-forming hexpeptides were identified by obtaining RI ≥ 7.

Accession number of Gene Ontology	Number of Proteins	Number of hexpeptides	Frequency of Predicted fibril-forming hexpeptides
Biological process	369935	131289447	0.12 ± 0.17
0065007	2963	1742818	0.10 ± 0.15
0009987	152145	50481819	0.12 ± 0.17
0032502	6179	3665307	0.09 ± 0.13
0051234	13655	6183514	0.13 ± 0.20
0002376	1070	442485	0.10 ± 0.14
0008152	151601	48557241	0.12 ± 0.17
0051704	1065	363310	0.10 ± 0.16
0032501	2209	1330066	0.10 ± 0.15
0048519	4002	1972548	0.09 ± 0.14
0048518	3638	1908650	0.09 ± 0.13
0050789	15286	7280097	0.10 ± 0.14
0050896	12705	5229936	0.11 ± 0.16
Cellular component	162913	69117049	0.12 ± 0.18
0044464	125461	50190897	0.13 ± 0.19
0032991	5476	2915149	0.10 ± 0.14
0043226	12336	6332565	0.10 ± 0.14
0044422	16365	7892350	0.11 ± 0.17
0044423	1082	429344	0.10 ± 0.15
Molecular function	251671	97593593	0.12 ± 0.17
0005488	93440	36212221	0.11 ± 0.16
0003824	131187	50137625	0.12 ± 0.18
0009055	1826	477887	0.16 ± 0.25
0030234	1107	631219	0.09 ± 0.14
0060089	2090	1299918	0.11 ± 0.16
0030528	4651	1815311	0.09 ± 0.13
0045182	4989	2029483	0.11 ± 0.17
0005215	10663	4173787	0.14 ± 0.21

## Discussion

Amyloid fibrillar aggregates of proteins or polypeptides are potentially lethal and related to many diseases, such as Alzheimer's disease, Parkinson's disease, transmissible spongiform encephalopathies, and type II diabetes [1-4]. Here, we have described a method for predicting the amyloid fibril-forming propensities of hexpeptides. Moreover, we used this approach to identify the possible fibril- forming peptides in all hexpeptides and the frequency of amyloid fibril-forming hexpeptides in proteins of the UniProt database. All of these results will help us in understanding the cause of the amyloid fibrillar aggregates of peptides.

In this study, we did not start from some properties which had been proved to relate with the amyloid fibrillar aggregates of peptides, such as the Packing Density, Hydrophobicity, Charge β-sheet propensity and so on [5,6,14,27]. However, we firstly evaluated every physicochemical property in the AAindex and found some physicochemical properties related to amyloid fibrillar aggregates of proteins or polypeptides. Secondly, the genetic algorithm was used to select a physicochemical property set. The results indicated that the selected properties could complement one another to yield a powerful and efficient predictor. In addition, a hexpeptide with every physicochemical property was encoded by three elements rather than the average value of the corresponding property. The performance of employing this encode scheme was better than using one element (data not shown). The detailed encode schemes was shown in the section of Methods.

The features of Pafig did not directly contain the structural features of the proteins. Thus, it is possible that some of the structure information was ignored by Pafig, which also existed as the protein aggregation and fibril forming factors [1,20]. However, the lack of structural information was likely overcome by the inclusion of different physicochemical properties in the Pafig [39]. Moreover, the sample size of the training dataset of Pafig compared with the number of all hexpeptides was very small, which would affect the performance of Pafig. Therefore, the future work is to collect more data by combining biological knowledge and related sources and add some structure feature into Pafig.

## Conclusion

In this study, we used 41 physicochemical properties to identify the specific regions (six consecutive residues) associated with amyloid fibrillar aggregates. As this method is computationally efficient and accurate, it can be used to analyze large systems, such as entire proteomics data. Moreover, we have found that the amino acids – alanine, phenylalanine, isoleucine, leucine and valine occurred at the higher frequencies and the amino acids – aspartic acid, glutamic acid, histidine, lysine, arginine and proline appeared with the lower frequencies in the predicted fibril-forming hexpeptides. The amyloid fibrillar aggregation propensity of membrane proteins was somewhat higher than other proteins.

## Availability and requirements

Project name: Pafig [30]

Operating systems: Windows

Programming language: Perl

License: GNU General Public License. This license allows the source code to be redistributed and/or modified under the terms of the GNU General Public License as published by the Free Software Foundation. The source code for the application is available at no charge.

Any restrictions to use by non-academics: None

## Competing interests

The authors declare that they have no competing interests.

## Authors' contributions

Jian Tian wrote the code of Pafig. Ningfeng Wu and Yunliu Fan supervised the work. Jian Tian, Ningfeng Wu, and Jun Guo were involved in the preparation of the manuscript. Jian Tian, Ningfeng Wu, Jun Guo and Yunliu Fan read and approved the manuscript.

## Supplementary Material

Additional file 1 **Table S1. The fibril-forming peptides**. The file can be viewed by the software word. Click here for file
