# Supplementary material for: Prediction of amyloid fibril-forming segments based on a support vector machine
Source: BMC Bioinformatics. 2009 Jan 30;10(Suppl 1):S45. doi: 10.1186/1471-2105-10-S1-S45 (PMC2648769; doi:10.1186/1471-2105-10-S1-S45)
Supplement: Additional file 1 — Table S1. The fibril-forming peptides. The file can be viewed by the software word. [file 1471-2105-10-S1-S45-S1.doc]

Table S1. The fibril-forming peptides

| Fibril-forming segment | Source protein | Reference |
| --- | --- | --- |
| SMVLFSSPPV | human prion protein | [1] |
| SSPPVILLIS | human prion protein | [1] |
| ILLISFLIFL | human prion protein | [1] |
| FLIFLIVG | human prion protein | [1] |
| NLKHQPGGGKVQIVYKPVDLSKVTSKCGSLGNIHHKPGGGQVE | t-Protein | [2] |
| NLKHQPGGGKVQIVYKEVD | t-Protein | [2] |
| GKVQIVYK | t-Protein | [2] |
| VQIVYK | t-Protein | [2] |
| VPHQKLVFFAEDVGS | Amyloid beta A4 peptide | [3, 4] |
| EQVTNVGGAVVTGVTAVA | Alpha synuclein | [5] |
| TVNGVGEVTATAVQGVAV | Alpha synuclein | [5] |
| VTNVGGAVVTGVTAVA | Alpha synuclein | [5] |
| GVVGWVKNTSKGTVTGQVQG | Acyl Phosphatase | [6] |
| DWSFYLLYYTEFTPTGKDEYA | β-microglobulin | [7, 8] |
| TKRPRFLYEIAMALNSD | 434 Cro repressor | [9] |
| VLSEGEWQLVLHVWAKVEA | Sperm whale myoglobin | [10] |
| EGEWQLVLHVWAKVEADVAGHGQDILIRLFK | Sperm whale myoglobin | [10] |
| SAPNLATLVKVTTNHFTHEEAMMD | Myohemerythrin | [11] |
| EVVPHKKMHKDFLEKIGGL | Myohemerythrin | [11] |
| MPEEELLNAPGETYVVTL | French bean Plastocyanin | [12] |
| GETYVVTL | French bean Plastocyanin | [12] |
| GTYSFYT | French bean Plastocyanin | [12] |
| GTVSFVTSPHQGAGMVGKVTVN | French bean Plastocyanin | [12] |
| LSQTFVYGGSRAKRNN | Bovine Pancreatic Trypsin Inhibitor (BPTI) | [13] |
| MKVIFLKDVKG | N-terminal domain of Ribosomal protein L9 | [14] |
| GYANNFLFKQG | N-terminal domain of Ribosomal protein L9 | [14] |
| QISFADYNLLDLLRIHQVLN | Glutathione S Transferase P domain II (Glutex) | [15] |
| DILTLLNSTNKDWWKVEVND | Spectrin SH3 | [16] |
| DWWKVEVNDRQGFVPA | Spectrin SH3 | [16] |
| DILTLLNSTNKDWWKVEVNDRQGFVPA | Spectrin SH3 | [16] |
| FVNVQAVKVFLESQGIAY | Ada-2h | [17] |
| FVNVEAVKAFLEAHGIAY | Ada-2h | [17] |
| STNVKTAFEMVILDIYNNV | Ara | [17] |
| TESKEKITQYIYHVLNGEIL | Com-A | [17] |
| AKKENIIAAAQAGASGY | P21-ras | [18] |
| PFTAATLEEKLNKIFEKLGMY | P21-ras | [18] |
| GVGKSALTIQLIQNHFVY | P21-ras | [18] |
| RQGVEDAFYTLVREIRQHK | P21-ras | [18] |
| VTIKANLIFANGFTQTAEFKG | PL B1 protein | [19] |
| KGTFEKATSEAYAYADTLKKDNGEY | PL B1 protein | [19] |
| GEYTVDVADKGYTLNIKFAGD | PL B1 protein | [19] |
| GEWTYDDATKTFTVTE | Protein G | [20] |
| DWSFYLLYYTEFTPTGKDEYA | b2m | [21] |
| SNFLNCYVSGFHPSDIEVDLLK | b2m | [22] |
| NHVTLSQ | b2m | [23] |
| NFGAILSS | Amylin | [24] |
| AFGAILSS | Amylin | [24] |
| NFAAILSS | Amylin | [24] |
| NFGAALSS | Amylin | [24] |
| NFGAIASS | Amylin | [24] |
| QRLANFLVH | Amylin | [25] |
| SNNFGAIL | Amylin | [26] |
| NFLVHSSNN | Amylin | [27] |
| KPFTARFEGRIFSRSDELRALITEITGE | Phage | [28] |
| KPFLARVEGRIFSRSDELRAYITAYTGE | Phage | [28] |
| KPFTARISGRLFSRSDELKTIIATITGE | Phage | [28] |
| KPYIARFEGRLFSRSDELRAVIEAHTGE | Phage | [28] |
| KPFIARFEGRLFSRSDELKAIIKELTGE | Phage | [28] |
| KPFLARFRGRIFSRSDELRTLIAAFTGE | Phage | [28] |
| VGGAVVTGV | AlphaSyn | [29] |
| VTGVTAVQKTV | AlphaSyn | [30] |
| CPLMVKVLDAV | TTR | [31] |
| YTIAALLSPYS | TTR | [32] |
| RVEKVAILGLMVLA | S6-mutant | [33] |
| SFNNGDCFILD | Gelsolin | [34] |
| NAGDVAFV | Lactoferrin | [35] |
| NFGSVQFV | Lactadherin | [36] |
| SFFSFLGEAFD | Serum A (48) | [37] |
| DCVNITIKQHTVTT | Prion protein | [38] |
| DIKIMERVVEQMCTTQY | Prion protein | [38] |
| AGAAAAGAVVGGLGG | Prion protein | [38] |
| MKHMAGAAAAGAVV | Prion protein | [38] |
| PQGGYQQYN | Sup35 | [39] |
| GNNQQNY | Sup35 | [40] |
| AEFHRWSSYMVYWK | AChe | [41] |
| EASNCFAIRHFENKFAVETLICSRTVKKNIIEEN | ABri | [42] |
| EASNCFAIRHFENKFAVETLICFNLFLNSQEKHY | ADan | [42] |
| VTVKVNAVKVTV | De novo design | [43] |
| KETAAAKFERQHMDSSTSAA | Ribonuclease A | [32] |
| IKYLEFISQAIIHVLHSR | Myoglobin | [44] |
| VQIVYK | Tau | [2] |
| MLSNTTAIAEAWARL | a-tubulin | [45] |
| QKLVFFAEDVGSNKGAIIGLMVGGVVIA | A beta protein | [46] |
| NFLVHSSNNFGAILSS | IAPP | [46] |
| SAMSRPIIHFGSDYEDRYYRENMHRYPN | Human prion | [46] |
| DCVNITIKQHTVTTTT | Human prion | [46] |
| RHFWQQDEPPQSPWDRVKDLATVYVDVLKDSGRDYVSQFEGSALGKQLNLKLLDNWDSVTSTFSKLREQLGPVTQEFWDNLEKETEGLRQEMS | Apolipoprotein A-I | [46] |
| CPLMVKVLDA | Transthyretin | [46] |
| YTIAALLSPYS | Transthyretin | [46] |
| SNFLNCYVSGFHPSDIEVDLL | b2-microglobulin | [46] |
| KDWSFYLLYYTE FTPTEKDEYACRVNHVTLSQPKIVKWDR | b2-microglobulin | [46] |
| RSWFSFLGEAY | Amyloid A protein (AA) | [46] |
| NFGSVQ | **Medin** | [46] |
| VFMKGLSKAKEGVVAA | NAC peptide of a-synuclein | [46] |
| KEGVLYVGSKTKEGVVHGVATVAEKTKEQVTNVGGAVVTGVTAVAQKTVEGAGSIAAATGFVKKDQLG | NAC peptide of a-synuclein | [46] |
| GYLTVAAVFR | b-tubulin | [45] |
| SYGGEGIGNVAVAGELPVAGKTAVAGRVPIIGAVGFGGPAGAAGAVSIAGR | Chorion A | [47] |
| GNLPFLGTAGVAGEFPTA | Chorion B | [48] |

References

1. Fernandez-Escamilla AM, Rousseau F, Schymkowitz J, Serrano L: **Prediction of sequence-dependent and mutational effects on the aggregation of peptides and proteins**. *Nat Biotechnol* 2004, **22**(10):1302-1306.

2. von Bergen M, Friedhoff P, Biernat J, Heberle J, Mandelkow EM, Mandelkow E: **Assembly of tau protein into Alzheimer paired helical filaments depends on a local sequence motif ((306)VQIVYK(311)) forming beta structure**. *Proc Natl Acad Sci U S A* 2000, **97**(10):5129-5134.

3. Tjernberg L, Hosia W, Bark N, Thyberg J, Johansson J: **Charge attraction and beta propensity are necessary for amyloid fibril formation from tetrapeptides**. *J Biol Chem* 2002, **277**(45):43243-43246.

4. Wood SJ, Wetzel R, Martin JD, Hurle MR: **Prolines and amyloidogenicity in fragments of the Alzheimer's peptide beta/A4**. *Biochemistry* 1995, **34**(3):724-730.

5. Bodles AM, Guthrie DJ, Harriott P, Campbell P, Irvine GB: **Toxicity of non-abeta component of Alzheimer's disease amyloid, and N-terminal fragments thereof, correlates to formation of beta-sheet structure and fibrils**. *Eur J Biochem* 2000, **267**(8):2186-2194.

6. Chiti F, Taddei N, Baroni F, Capanni C, Stefani M, Ramponi G, Dobson CM: **Kinetic partitioning of protein folding and aggregation**. *Nat Struct Biol* 2002, **9**(2):137-143.

7. Busch A, Engemann S, Lurz R, Okazawa H, Lehrach H, Wanker EE: **Mutant huntingtin promotes the fibrillogenesis of wild-type huntingtin: a potential mechanism for loss of huntingtin function in Huntington's disease**. *J Biol Chem* 2003, **278**(42):41452-41461.

8. Trinh CH, Smith DP, Kalverda AP, Phillips SE, Radford SE: **Crystal structure of monomeric human beta-2-microglobulin reveals clues to its amyloidogenic properties**. *Proc Natl Acad Sci U S A* 2002, **99**(15):9771-9776.

9. Padmanabhan S, Jimenez MA, Rico M: **Folding propensities of synthetic peptide fragments covering the entire sequence of phage 434 Cro protein**. *Protein Sci* 1999, **8**(8):1675-1688.

10. Reymond MT, Merutka G, Dyson HJ, Wright PE: **Folding propensities of peptide fragments of myoglobin**. *Protein Sci* 1997, **6**(3):706-716.

11. Dyson HJ, Merutka G, Waltho JP, Lerner RA, Wright PE: **Folding of peptide fragments comprising the complete sequence of proteins. Models for initiation of protein folding. I. Myohemerythrin**. *J Mol Biol* 1992, **226**(3):795-817.

12. Dyson HJ, Sayre JR, Merutka G, Shin HC, Lerner RA, Wright PE: **Folding of peptide fragments comprising the complete sequence of proteins. Models for initiation of protein folding. II. Plastocyanin**. *J Mol Biol* 1992, **226**(3):819-835.

13. Kemmink J, Creighton TE: **Local conformations of peptides representing the entire sequence of bovine pancreatic trypsin inhibitor and their roles in folding**. *J Mol Biol* 1993, **234**(3):861-878.

14. Luisi DL, Wu WJ, Raleigh DP: **Conformational analysis of a set of peptides corresponding to the entire primary sequence of the N-terminal domain of the ribosomal protein L9: evidence for stable native-like secondary structure in the unfolded state**. *J Mol Biol* 1999, **287**(2):395-407.

15. Dragani B, Cocco R, Principe DR, Paludi D, Aceto A: **Conformational properties of five peptides corresponding to the entire sequence of glutathione transferase domain II**. *Arch Biochem Biophys* 2001, **389**(1):15-21.

16. Viguera AR, Jimenez MA, Rico M, Serrano L: **Conformational analysis of peptides corresponding to beta-hairpins and a beta-sheet that represent the entire sequence of the alpha-spectrin SH3 domain**. *J Mol Biol* 1996, **255**(3):507-521.

17. Munoz V, Blanco FJ, Serrano L: **The distribution of alpha-helix propensity along the polypeptide chain is not conserved in proteins from the same family**. *Protein Sci* 1995, **4**(8):1577-1586.

18. Munoz V, Serrano L, Jimenez MA, Rico M: **Structural analysis of peptides encompassing all alpha-helices of three alpha/beta parallel proteins: Che-Y, flavodoxin and P21-ras: implications for alpha-helix stability and the folding of alpha/beta parallel proteins**. *J Mol Biol* 1995, **247**(4):648-669.

19. Ramirez-Alvarado M, Serrano L, Blanco FJ: **Conformational analysis of peptides corresponding to all the secondary structure elements of protein L B1 domain: secondary structure propensities are not conserved in proteins with the same fold**. *Protein Sci* 1997, **6**(1):162-174.

20. Blanco FJ, Serrano L: **Folding of protein G B1 domain studied by the conformational characterization of fragments comprising its secondary structure elements**. *Eur J Biochem* 1995, **230**(2):634-649.

21. Jones S, Manning J, Kad NM, Radford SE: **Amyloid-forming peptides from beta2-microglobulin-Insights into the mechanism of fibril formation in vitro**. *J Mol Biol* 2003, **325**(2):249-257.

22. Kozhukh GV, Hagihara Y, Kawakami T, Hasegawa K, Naiki H, Goto Y: **Investigation of a peptide responsible for amyloid fibril formation of beta 2-microglobulin by achromobacter protease I**. *J Biol Chem* 2002, **277**(2):1310-1315.

23. Ivanova MI, Sawaya MR, Gingery M, Attinger A, Eisenberg D: **An amyloid-forming segment of beta2-microglobulin suggests a molecular model for the fibril**. *Proc Natl Acad Sci U S A* 2004, **101**(29):10584-10589.

24. Azriel R, Gazit E: **Analysis of the minimal amyloid-forming fragment of the islet amyloid polypeptide. An experimental support for the key role of the phenylalanine residue in amyloid formation**. *J Biol Chem* 2001, **276**(36):34156-34161.

25. Jaikaran ET, Higham CE, Serpell LC, Zurdo J, Gross M, Clark A, Fraser PE: **Identification of a novel human islet amyloid polypeptide beta-sheet domain and factors influencing fibrillogenesis**. *J Mol Biol* 2001, **308**(3):515-525.

26. Kapurniotu A, Schmauder A, Tenidis K: **Structure-based design and study of non-amyloidogenic, double N-methylated IAPP amyloid core sequences as inhibitors of IAPP amyloid formation and cytotoxicity**. *J Mol Biol* 2002, **315**(3):339-350.

27. Mazor Y, Gilead S, Benhar I, Gazit E: **Identification and characterization of a novel molecular-recognition and self-assembly domain within the islet amyloid polypeptide**. *J Mol Biol* 2002, **322**(5):1013-1024.

28. Koscielska-Kasprzak K, Otlewski J: **Amyloid-forming peptides selected proteolytically from phage display library**. *Protein Sci* 2003, **12**(8):1675-1685.

29. Du HN, Tang L, Luo XY, Li HT, Hu J, Zhou JW, Hu HY: **A peptide motif consisting of glycine, alanine, and valine is required for the fibrillization and cytotoxicity of human alpha-synuclein**. *Biochemistry* 2003, **42**(29):8870-8878.

30. Giasson BI, Murray IV, Trojanowski JQ, Lee VM: **A hydrophobic stretch of 12 amino acid residues in the middle of alpha-synuclein is essential for filament assembly**. *J Biol Chem* 2001, **276**(4):2380-2386.

31. Gustavsson A, Engstrom U, Westermark P: **Normal transthyretin and synthetic transthyretin fragments form amyloid-like fibrils in vitro**. *Biochem Biophys Res Commun* 1991, **175**(3):1159-1164.

32. Thompson MJ, Sievers SA, Karanicolas J, Ivanova MI, Baker D, Eisenberg D: **The 3D profile method for identifying fibril-forming segments of proteins**. *Proc Natl Acad Sci U S A* 2006, **103**(11):4074-4078.

33. Otzen DE, Kristensen O, Oliveberg M: **Designed protein tetramer zipped together with a hydrophobic Alzheimer homology: a structural clue to amyloid assembly**. *Proc Natl Acad Sci U S A* 2000, **97**(18):9907-9912.

34. Maury CP, Liljestrom M, Boysen G, Tornroth T, de la Chapelle A, Nurmiaho-Lassila EL: **Danish type gelsolin related amyloidosis: 654G-T mutation is associated with a disease pathogenetically and clinically similar to that caused by the 654G-A mutation (familial amyloidosis of the Finnish type)**. *J Clin Pathol* 2000, **53**(2):95-99.

35. Nilsson MR, Dobson CM: **In vitro characterization of lactoferrin aggregation and amyloid formation**. *Biochemistry* 2003, **42**(2):375-382.

36. Haggqvist B, Naslund J, Sletten K, Westermark GT, Mucchiano G, Tjernberg LO, Nordstedt C, Engstrom U, Westermark P: **Medin: an integral fragment of aortic smooth muscle cell-produced lactadherin forms the most common human amyloid**. *Proc Natl Acad Sci U S A* 1999, **96**(15):8669-8674.

37. Westermark GT, Engstrom U, Westermark P: **The N-terminal segment of protein AA determines its fibrillogenic property**. *Biochem Biophys Res Commun* 1992, **182**(1):27-33.

38. Gasset M, Baldwin MA, Lloyd DH, Gabriel JM, Holtzman DM, Cohen F, Fletterick R, Prusiner SB: **Predicted alpha-helical regions of the prion protein when synthesized as peptides form amyloid**. *Proc Natl Acad Sci U S A* 1992, **89**(22):10940-10944.

39. Patino MM, Liu JJ, Glover JR, Lindquist S: **Support for the prion hypothesis for inheritance of a phenotypic trait in yeast**. *Science* 1996, **273**(5275):622-626.

40. Balbirnie M, Grothe R, Eisenberg DS: **An amyloid-forming peptide from the yeast prion Sup35 reveals a dehydrated beta-sheet structure for amyloid**. *Proc Natl Acad Sci U S A* 2001, **98**(5):2375-2380.

41. Cottingham MG, Hollinshead MS, Vaux DJ: **Amyloid fibril formation by a synthetic peptide from a region of human acetylcholinesterase that is homologous to the Alzheimer's amyloid-beta peptide**. *Biochemistry* 2002, **41**(46):13539-13547.

42. Vidal R, Revesz T, Rostagno A, Kim E, Holton JL, Bek T, Bojsen-Moller M, Braendgaard H, Plant G, Ghiso J *et al*: **A decamer duplication in the 3' region of the BRI gene originates an amyloid peptide that is associated with dementia in a Danish kindred**. *Proc Natl Acad Sci U S A* 2000, **97**(9):4920-4925.

43. Orpiszewski J, Benson MD: **Induction of beta-sheet structure in amyloidogenic peptides by neutralization of aspartate: a model for amyloid nucleation**. *J Mol Biol* 1999, **289**(2):413-428.

44. Fandrich M, Forge V, Buder K, Kittler M, Dobson CM, Diekmann S: **Myoglobin forms amyloid fibrils by association of unfolded polypeptide segments**. *Proc Natl Acad Sci U S A* 2003, **100**(26):15463-15468.

45. Baumann MH, Wisniewski T, Levy E, Plant GT, Ghiso J: **C-terminal fragments of alpha- and beta-tubulin form amyloid fibrils in vitro and associate with amyloid deposits of familial cerebral amyloid angiopathy, British type**. *Biochem Biophys Res Commun* 1996, **219**(1):238-242.

46. Zhang Z, Chen H, Lai L: **Identification of amyloid fibril-forming segments based on structure and residue-based statistical potential**. *Bioinformatics* 2007, **23**(17):2218-2225.

47. Iconomidou VA, Vriend G, Hamodrakas SJ: **Amyloids protect the silkmoth oocyte and embryo**. *FEBS Lett* 2000, **479**(3):141-145.

48. Iconomidou VA, Chryssikos GD, Gionis V, Vriend G, Hoenger A, Hamodrakas SJ: **Amyloid-like fibrils from an 18-residue peptide analogue of a part of the central domain of the B-family of silkmoth chorion proteins**. *FEBS Lett* 2001, **499**(3):268-273.
